# Fidelibacter multiformis gen. nov., sp. nov., isolated from a deep subsurface aquifer and proposal of Fidelibacterota phyl. nov., formerly called Marine Group A, SAR406 or Candidatus Marinimicrobia

**DOI:** 10.1099/ijsem.0.006558

**Published:** 2024-10-25

**Authors:** Taiki Katayama, Masaru K. Nobu, Yoichi Kamagata, Hideyuki Tamaki

**Affiliations:** 1 Research Institute for Geo-Resources and Environment, Geological Survey of Japan (GSJ), National Institute of Advanced Industrial Science and Technology (AIST), Tsukuba, 305-8567, Japan; 2 Institute for Extra-cutting-edge Science and Technology Avant-garde Research (X-star), Japan Agency for Marine-Earth Science and Technology (JAMSTEC), Yokosuka, 237-0061, Japan; 3 Bioproduction Research Institute, National Institute of Advanced Industrial Science and Technology, Tsukuba, 305-8566, Japan

**Keywords:** auxotrophy, deep biosphere, energy-saving, Marine Group A, Marinimicrobia, peptidoglycan

## Abstract

A Gram-negative, obligatory anaerobic, chemoheterotrophic bacterium, designated strain IA91^T^, was isolated from sediments and formation water from deep aquifers in Japan. IA91^T^ derives its peptidoglycan, energy and carbon from exogenous cell wall fragments, namely muropeptides, released from actively reproducing bacteria, and is dependent on other bacteria for cell wall formation, growth and even cell shape: IA91^T^ is irregular rod-shaped but coccoids when muropeptide is absent. IA91^T^ grew in a temperature range of 25–45 °C with optimum growth at 40 °C. IA91^T^ utilized limited substrates, yeast extract, muropeptides and d-lactate. The major end products from yeast extract degradation were acetate, hydrogen and carbon dioxide. Co-cultivation with a hydrogen-scavenging methanogenic archaeon promoted IA91^T^ growth. No anaerobic respiration with nitrate, nitrite, sulphate or Fe(III) was observed. The major cellular fatty acids are C16 : 0, C18 : 1 *trans*9, C18 : 0 and C17 : 0. The G+C content of the genomic DNA was 45.6 mol%. Phylogenetic analysis based on 16S rRNA gene and conserved protein sequences involved in replication, transcription and translation indicated that IA91^T^ belonged to the candidate phylum Marine Group A (MG-A, SAR406 or *Ca*. Marinimicrobia) with no cultivated representatives. Based on the phenotypic and phylogenomic characteristics, a new genus and species, *Fidelibacter multiformis* gen. nov., sp. nov., is proposed for IA91^T^ (= JCM 39387^T^ = KCTC 25736^T^). In addition, a new bacterial phylum named *Fidelibacterota* phyl. nov. is proposed for the candidate phylum MG-A represented by *F. multiformis* and *Fidelibacteraceae* fam. nov., *Fidelibacterales* ord. nov. and *Fidelibacteria* classis nov.

## Introduction

A majority of Earth’s prokaryotes live under energy-limited conditions, including extremely low fluxes of organics in the deep subsurface and oxygen deprivation in ‘anaerobic’ ecosystems (much of which overlaps) [1,3]. While organisms in such energy-limited environments are thought to play critical roles in carbon and nutrient cycling, most remain uncultured [4,5], leaving many knowledge gaps. Energy limitation is known to drive genome-streamlining, cell size reduction and even ecological dependencies, such as nutrient exchange and division of labour [6,8], which may hinder efforts in cultivation, as also reflected in our studies on the isolation of uncharted lineages [9,10].

Recently, we have also succeeded in isolating a bacterium, designated strain IA91^T^, belonging to the candidate division Marine Group A [11] [MG-A; also known as SAR406 [12], *Ca*. Marinimicrobia [13], *Ca*. Neomarinimicrobiota (in NCBI taxonomy) or *Ca*. Marinisomatota (in Genome Taxonomy Database [GTDB] release 207 [14])] from an anaerobic deep subsurface aquifer [15]. The members of MG-A are widely distributed in ocean, freshwater, subsurface and engineered environments and may participate in nitrogen and sulphur cycling and show signs of genome-streamlining indicative of adaptation to energy-limited habitats [16]. Intriguingly, IA91^T^ exhibited an energy-saving strategy involving loss of the ability to independently form its own cell wall and instead assimilating peptidoglycan (PG)-recycling intermediates, muropeptides, derived from other growing bacteria [15]. Additionally, reliance on exogenous muropeptides can be traced to the ancestors of the MG-A phylum and vertically inherited by anaerobic sub-lineages [15]. In this study, we report the taxonomic characterization of IA91 based on morphological, physiological and genomic analyses.

## Methods

### Isolation and cultivation

Environmental sample collection and isolation of IA91^T^ were previously performed [15]. Briefly, sediment and formation water samples were collected from a commercial natural gas and water-producing well in Chiba prefecture, Japan (35.41 N 140.35 E). These samples were derived from gas-bearing aquifers deposited in deep marine environments during the Plio-Pleistocene period. The environmental conditions were a water temperature of 24.4 °C, pH 7.7 and redox potential of −213 mV, with a Cl^−^ concentration of 17 000 mg l^−1^. Slurries composed of sediment and formation water samples were incubated without additional nutrients under an N_2_/CO_2_ (80 : 20) atmosphere at 45 °C. Methane-producing cultures obtained were inoculated into a saline mineral medium [17] amended with 0.2 g l^−1^ Bacto peptone, 0.2 g l^−1^ yeast extracts, 1 mM pyruvate, 2 mM coenzyme M, 0.1 mM titanium (III) citrate and 0.2 ml aliquots of the culture of a *Bacillota* strain Acc8 (=JCM 39386) separately isolated from the same environment. This setup was designed to cultivate microorganisms interacting with Acc8 (for details, see [15]). The isolation of IA91^T^ was achieved using the deep agar slant method combined with the dilution-to-extinction method [17]. The pure culture of IA91^T^ was incubated under an N_2_/CO_2_ (95 : 5) atmosphere at 40 °C in saline mineral medium (pH 7.8) amended with 5.0 g l^−1^ yeast extract, 0.3 g l^−1^ Na_2_S⋅9H_2_O, 0.3 g l^−1^ l-cysteine⋅HCl and either (i) 330 ml l^−1^ autoclaved Acc8 culture supernatant (presumably containing Acc8-derived muropeptides derived from strain Acc8) or (ii) muropeptides obtained by the enzymatic digestion of PG from *Bacillus subtilis* (Sigma-Aldrich, Japan) [15], unless otherwise indicated.

### Phenotypic characterization

Cells grown in the saline mineral medium supplemented with autoclaved Acc8 culture supernatant at 40°C were harvested during the mid-exponential growth phase and observed for cell morphology using phase-contrast and fluorescence microscopy (BX51; Olympus, Japan).

All physiological and chemotaxonomic experiments were performed in triplicate. The end products of yeast extract (5.0 g l^−1^) degradation were analysed for IA91 pure culture by high-performance liquid chromatography using an RSpak KC-811 column (Shodex; eluent, 3 mM HClO_4_; column temperature, 50 °C) and a UV detector (210 nm, Shimadzu SPD-10A) and gas chromatography as described below. To assess the effect of temperature, pH, NaCl concentration or antibiotics on IA91 growth, H_2_ concentration in the gas phase of the IA91 pure culture was measured as a growth indicator. Due to the poor growth, optical density measurements were unsuitable for evaluating and comparing IA91 growth under different culture conditions. For the oxygen tolerance and anaerobic respiration tests, IA91 growth was determined via quantitative PCR targeting the 16S rRNA gene of IA91 (see [15] for details). In the substrate utilization experiments, IA91 was co-cultured with *Methanothermobacter thermautotrophicus* ∆H, and its growth was assessed by a significant increase in CH_₄_ production compared to the negative control (medium supplemented with autoclaved Acc8 culture supernatant and 2.0 g L⁻¹ yeast extract). The co-culture system was employed to clarify the difference in IA91 growth, as CH_₄_ production in co-culture is substantially higher than H_₂_ production in pure culture [15]. H_2_, CH_4_ and CO_2_ were measured using gas chromatography (GC-2014AT; Shimadzu, Japan) equipped with an active carbon column (mesh range 60/80) maintained at 100 °C. Gas components were separated using argon as the carrier gas and quantified using a thermal conductivity detector, following calibration with standard gases.

Antibiotic sensitivity was examined using the following compounds (each at 50 mg l^−1^): ampicillin, chloramphenicol, kanamycin, neomycin, rifampicin and vancomycin. Oxygen tolerance was evaluated by culturing IA91 in three different cultures: medium with or without reducing agents under an N_2_/CO_2_ (95 : 5) atmosphere or medium without reducing agents under an atmospheric condition. Anaerobic respiration capabilities were examined using nitrate, nitrite, sulphate or Fe(III) (each at 8 mM) in the pure culture supplemented with 5.0 g l^−1^ yeast extract, autoclaved Acc8 culture supernatant and 8 mM d-lactate. Substrate utilization was tested with the following substrates (each at 8 mM): pyruvate, glucose, fructose, galactose, mannose, ribose, xylose, arabinose, sucrose, lactose, starch, cellulose, mannitol, sorbitol, raffinose, cellobiose or d-lactate.

For the fatty acid analysis, cells grown in the saline mineral medium amended with autoclaved Acc8 culture supernatant at 40 °C were harvested during the late exponential phase. Fatty acid methyl esters were prepared and analysed using the protocol of the Sherlock Microbial Identification System version 6.0 (Microbial ID; MIDI Inc., USA) and TSBA library database (TSBA6 6.20).

### Genomic and phylogenetic analysis

The complete genome sequence of IA91^T^ was previously determined and annotated [15]. A maximum-likelihood tree based on the 16S rRNA gene sequences was constructed using sequences from related type strains belonging to the phyla *Calditrichota*, *Bacteroidota*, *Ignavibacteriota*, *Chlorobiota*, *Fibrobacterota*, *Gemmatimonadota*, *Planctomycetota*, *Chlamydiota* and *Verrucomicrobiota*. Sequences were aligned using the silva v138.2 database with mothur v.1.48 [18]. The maximum-likelihood tree was reconstructed using IQ-TREE v2.2.6 [19], applying an optimal model chosen by ModelFinder (-m MFP) and 1000 ultrafast bootstrap replicates. Transfer bootstrap branch support values were calculated using BOOSTER v0.1.2 [20]. Genome sequences of related species and metagenome-assembled genomes (MAGs) with≥85% completeness and≤5% contamination were obtained from GTDB release 09-RS220. Conserved marker protein sequences involved in replication, transcription and translation (Table S1, available in the online version of this article) are aligned with MAFFT v7.49 [21], concatenated and trimmed with BMGE v1.12 (-g 0.67 m BLOSUM30 -b 3) [22]. A maximum-likelihood tree was constructed using IQ-TREE v2.2.6 (-m Poisson+UDM0064 LCLR) [19] with 1000 ultrafast bootstrap replicates. Transfer bootstrap branch support values were calculated with BOOSTER v0.1.2 [20].

For the calculation of average nucleotide identity (ANI) between MG-A and related type strains, PYANI v.1.3 [23] using BLADTN+ (-m ANIb) was performed. To calculate pairwise distances between MG-A and related phyla, aligned 16S rRNA sequences were obtained from the silva v138.2 database [24]. Sequences longer than 1300 bp were retained and clustered at 97% sequence identity using CD-HIT v4.8.1 [25]. Distances between the representative sequences were calculated with mothur v.1.48 using the dist.seq function (no penalization for gaps; calc = nogaps, countends = F). Statistical significance (*P*-values) was determined using Student’s t-test.

The environmental origin of clones that are closely related to IA91^T^ (≥99 or ≥97 % sequence similarities in 16S rRNA gene) was searched against 16S rRNA gene amplicon datasets available in Sequence Read Archive using the IMNGS v1.0 [26].

## Results and discussion

### Morphology and physiology

**Fig. 1.**
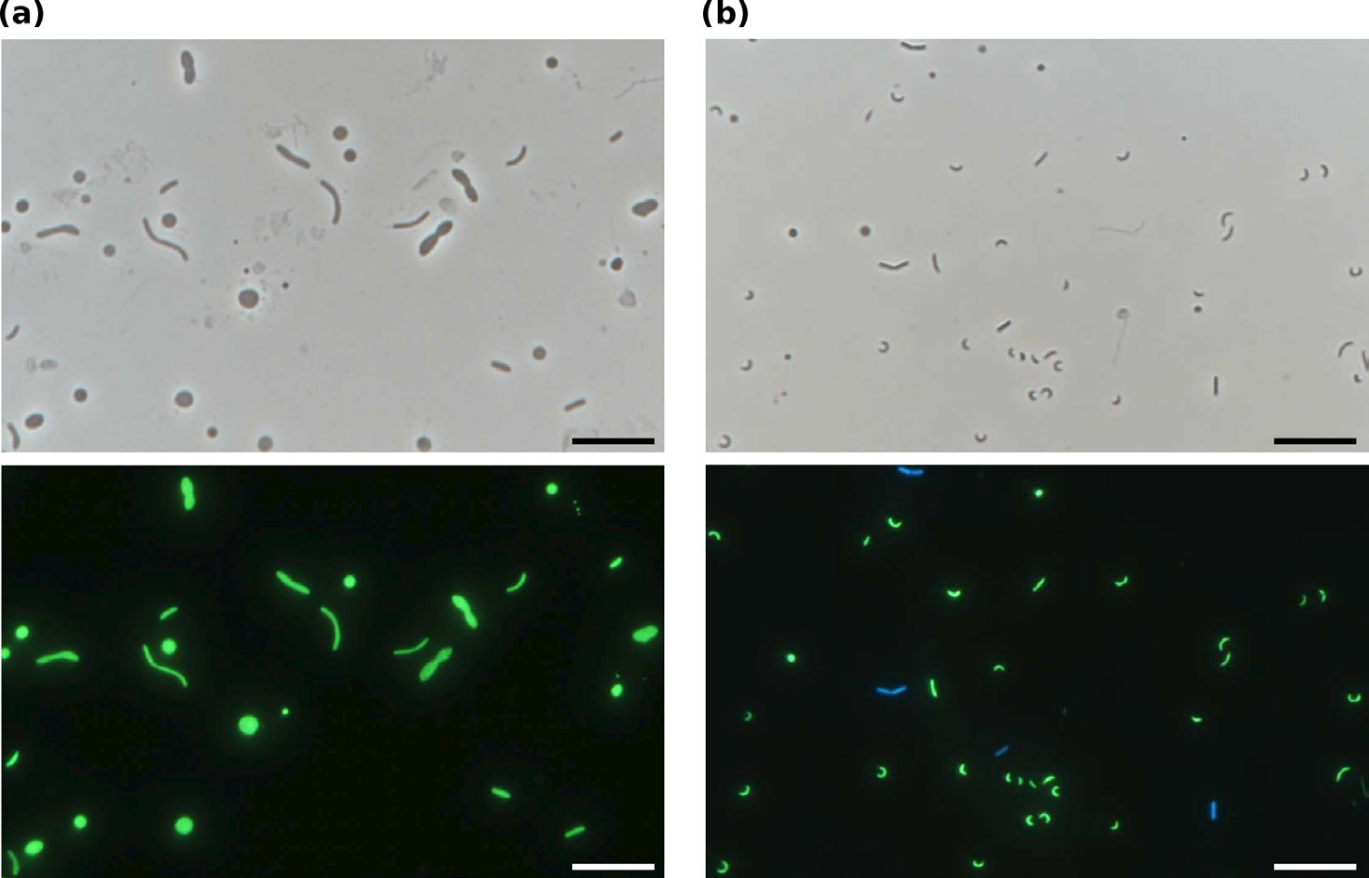
Representative phase-contrast micrographs of IA91^T^ cells co-cultured with a hydrogen-scavenging methanogen. Cultures supplemented with muropeptide (**a**) or muropeptide plus other PG components (**b**). Cell were stained with SYBR green I (lower pictures). The methanogen cells were visible in cyan due to their autofluorescence (lower pictures). Scale bars, 10 µm.

As previously reported [15], IA91^T^ cells were Gram-stain negative, non-spore-forming and non-motile. Cryo-electron microscopy revealed the presence of outer and inner membranes as well as a lipopolysaccharide structure on the cell surface [15]. The cells exhibited irregular rod shapes in the presence of muropeptide but formed coccoid shapes in its absence due to the inability of IA91^T^ to synthesize PG [15]. The length and width of the rod-shaped cells and the diameter of the spherical cells varied with culture conditions (Fig. 1a). In cultures supplemented with muropeptides and other PG components that IA91^T^ cannot synthesize – namely, *N*-acetylmuramic acid (MurNAc), d-alanine, d-glutamic acid, diaminopimelic acid and lysine – the cells exhibited thin, short, curved-rod shapes (Fig. 1b). Colonies of IA91^T^ appeared as white, slightly irregular discs with wavy margins on deep agar slants after 1 month of incubation at 40 °C.

IA91^T^ required yeast extract for growth in addition to a source of muropeptides and neither peptone nor casamino acids stimulated growth. The end products of yeast extract degradation in pure culture are acetate (0.4 ± 0.04 mM), hydrogen (1.2 ± 0.05 mM) and carbon dioxide (1.5 ± 0.1 mM) (mean ± sd). The co-culture with hydrogen-scavenging methanogen stimulated IA91^T^ growth [15]. IA91^T^ grew within a temperature range of 25–45 °C, with optimal growth at 40 °C (Fig. S1a). No growth at 20 or 50 °C. At 25 °C, growth was detected after 4 months of incubation. IA91^T^ grew at a pH range of 6.8–8.5, with optimal growth at pH 7.8 (Fig. S1b). No growth was observed at pH 6.5 or 9.0. IA91^T^ grew in a salinity range of 0.05–1.2 M NaCl, with optimal growth occurring at 0.3–0.4 M (Fig. S1c). No growth was observed in 0 or 1.5 M NaCl. In cultures with≤0.1 M or≥1.2 M NaCl, total amounts of produced H_2_ were lower by>80 % compared to optimal conditions after 3 months of incubations. The addition of neomycin did not affect IA91^T^ growth, whereas growth was almost completely inhibited by chloramphenicol, vancomycin and rifampicin (Fig. S1d). The presence of kanamycin and ampicillin slightly inhibited the growth. In the oxygen tolerance test, reducing agents were required for growth (Supplementary Fig. S2a). In the anaerobic respiration test, no growth was observed in the presence of nitrate, nitrite, sulphate or Fe(III) as an electron donor (Fig. S2b). In the substrate utilization text, significant CH_4_ production was observed only in the cultures amended with d-lactate (Fig. S3). The addition of other tested substrates did not proceed with CH_4_ production compared to cultures without substrate addition (Fig. S3).

The amino acids of cell-wall murein were previously qualitatively determined to include alanine, glutamic acid and lysin [15]. The major cellular fatty acids of IA91^T^ were C16 : 0 (48.7%), C18 : 1 *trans*9 (25.2%), C18 : 0 (6.3%), C17 : 0 (6.1%), C16 : 1 *cis*9 (4.1%), C17 : 1 *cis*10 (3.2%), C15 : 0 (2.6%), C18 : 1 *cis*9 (2.1%) and C19 : 1 *cis*10 (1.6%).

### Genomics

**Fig. 2.**
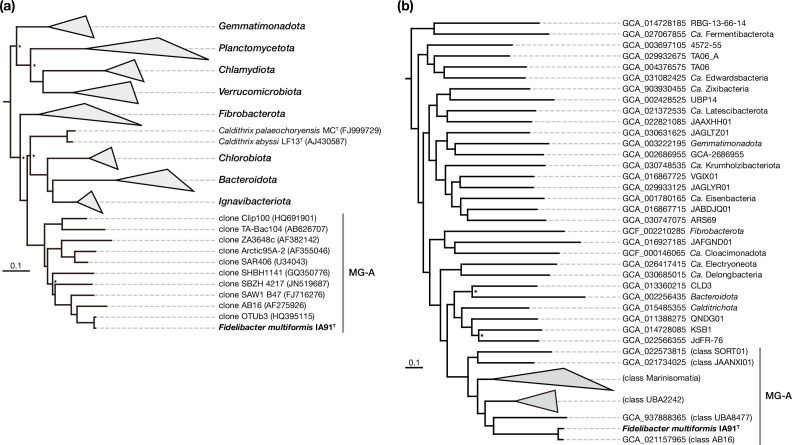
A maximum-likelihood tree showing the relationship of strain IA91^T^ (bold), MG-A members and other members of the bacterial phyla based on 16S rRNA genes (**a**) and conserved marker proteins involved in replication, transcription and translation (**b**). Branches with lower bootstrap values (<95 %) are indicated by asterisk. In (**b**), a partial tree is shown. The entire tree is depicted in Fig. S4.

**Fig. 3.**
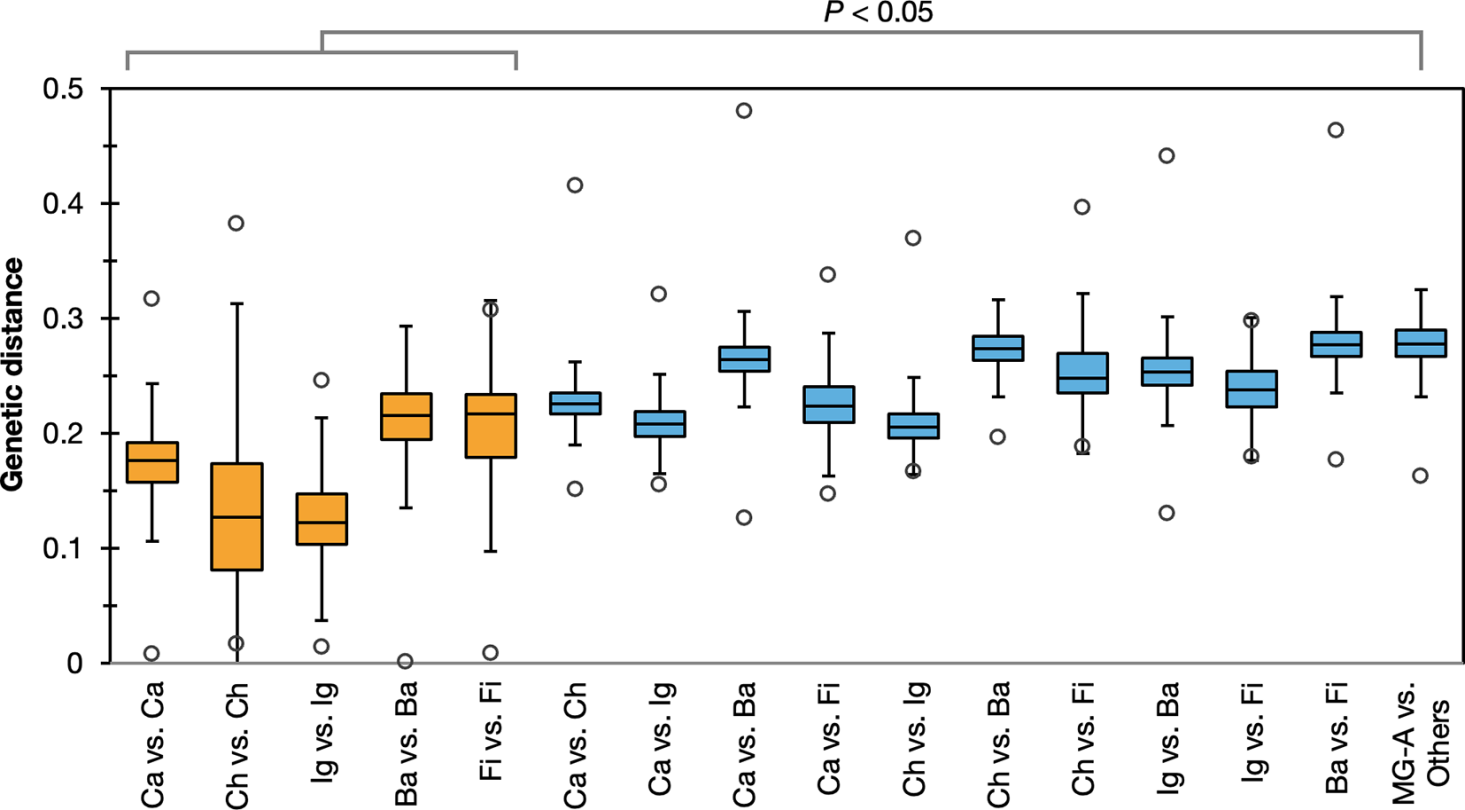
Box plots of the genetic distances between MG-A and related bacterial phyla. Centre lines indicate the median, box limits indicate the first and third quartiles, whiskers indicate 1.5× interquartile range and dots indicate the minimum and maximum values. Statistical significance is indicated for comparison of the distances between phyla. Ca, *Calditrichota*; Ch, *Chlorobiota*; Ig, *Ignavibacteriota*; Ba, *Bacteroidota*; Fi, *Fibrobacterota*.

The IA91^T^ genome size was 2.79 Mbp with a DNA G+C content of 45.6 mol%. The genome was estimated to contain 2290 protein-coding genes. In 16S rRNA gene sequences, the closest cultivated species to IA91^T^ was *Calorithrix insularis* in the phylum *Calditrichota*, sharing 83.8% sequence similarity. Phylogenetic trees based on 16S rRNA gene sequences and marker proteins showed that IA91^T^ belongs to MG-A, forming a distinct clade apart from other bacterial phyla (Figs 2 and S4). The ANI values between IA91^T^ and the type strains of related phyla (*Calditrichota*, *Chlorobiota*, *Ignavibacteriota*, *Bacteroidota* and *Fibrobacterota*) were below 75% (Table S2), indicating that IA91^T^ represents a novel species. To assess if MG-A represents a distinct bacterial phylum, we calculated pairwise distances based on 16S rRNA gene sequences. These distances between MG-A and related phyla (*Calditrichota*, *Chlorobiota*, *Ignavibacteriota*, *Bacteroidota* and *Fibrobacterota*) were significantly greater (*P* < 0.05) than the distances within these phyla (Fig. 3).

### Ecology

Our previous study indicated that IA91^T^ utilized exogenous muropeptide not only for PG synthesis but also as energy and carbon sources [15]. During muropeptide catabolism, the conversion of MurNAc-6P into *N*-acetylglucosamine-6P yields d-lactate, which is then oxidized to pyruvate by d-lactate dehydrogenase [15]. In this study, we found that IA91^T^ utilized only d-lactate but not sugars, peptides (peptone) and amino acids (casamino acids). This limited substrate utilization further emphasizes IA91^T^’s dependence on muropeptide for energy and carbon sources in natural settings. To assess whether d-lactate utilization is also found in related taxa, the gene coding for d-lactate dehydrogenase was searched against related MAGs. According to GTDB taxonomy, IA91^T^ belonged to the candidate class AB16 and genus 46–47. As previously reported [15], all members of class AB16 lacked the genes required for PG synthesis but possessed the genes involved in muropeptide recycling, suggesting that AB16 bacteria require exogenous PG fragments for cell wall synthesis, similar to IA91^T^. Among the genus 46–47, three candidate species, 46–47 (including IA91), M50B103 and TCS52, were recognized in GTDB. The d-lactate dehydrogenase gene was detected in most members of 46–47 and M50B103, but not in TCS52. This suggests that energy-saving strategies involving PG auxotrophy may be slightly different among these species.

**Fig. 4.**
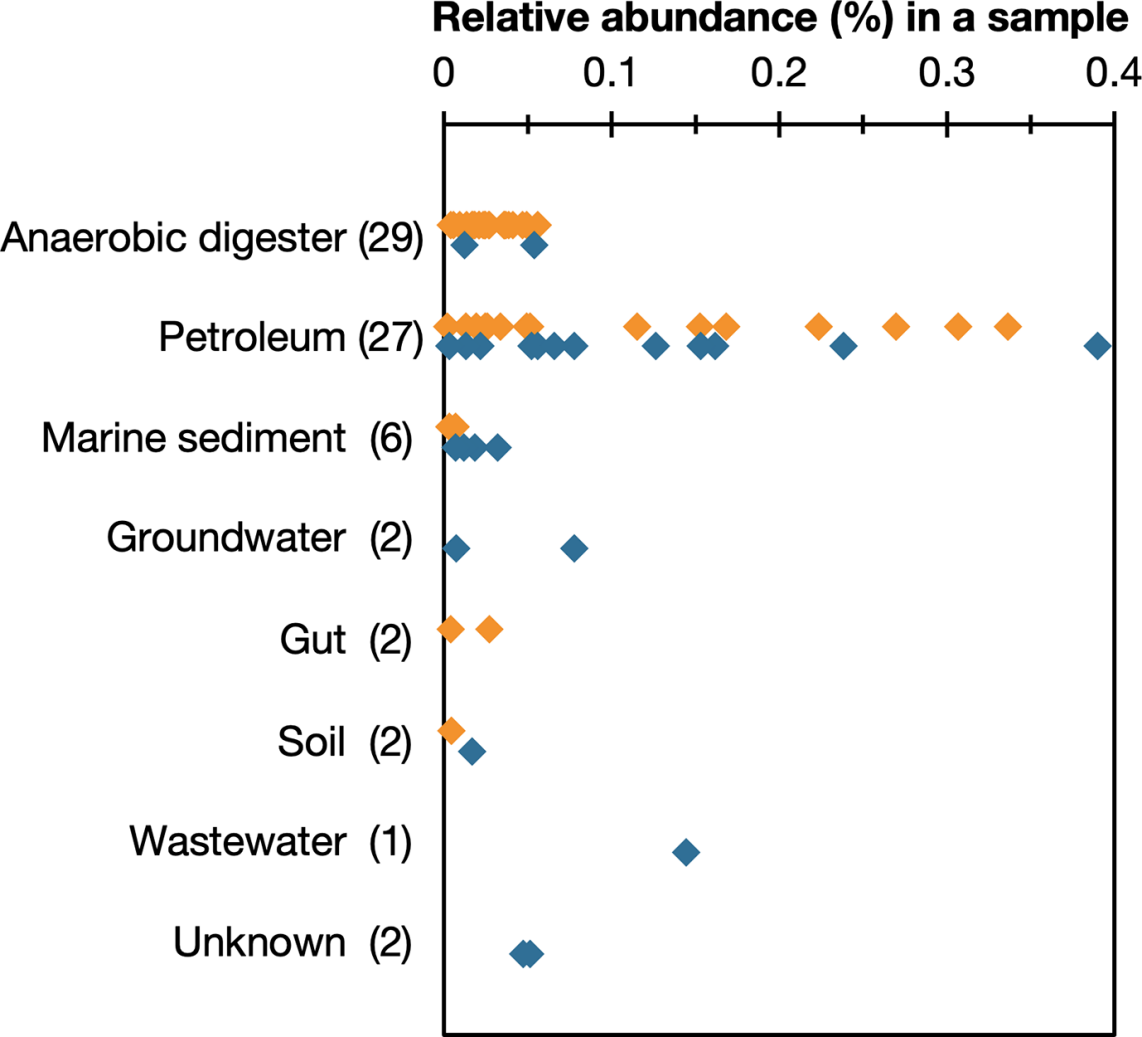
Environmental distribution of IA91^T^-related sequences in the 16S rRNA gene amplicon database. The sequences that showed ≥99% or 97–99% similarity with IA91^T^ are indicated by orange or blue diamond, respectively. The number in parenthesis represents the number of environmental samples, in which the sequences were detected.

Based on publicly available 16S rRNA gene amplicon datasets, IA91^T^-related populations (≥97% sequence similarity) were detected as a rare biosphere (≤0.4% relative abundance) in anoxic, organic-rich environments with high microbial densities where fermentation and methanogenesis generally occur, such as anaerobic digesters, petroleum reservoirs and marine sediments (Fig. 4). These environments are consistent with the physiology of IA91^T^, which exhibits a PG-auxotrophic, fermentative and syntrophic lifestyle.

Based on unique phenotypic, genotypic and phylogenetic characteristics, we propose strain IA91^T^ (=JCM 39387^T^=KCTC 25736^T^) as a new species, *Fidelibacter multiformis* gen. nov., sp. nov. In addition, based on the phylogenomic analysis, we further propose a new phylum *Fidelibacterota* phyl. nov. Note that retaining the original candidate phylum name *Ca*. Marinimicrobia is inappropriate because the genus name *Marinimicrobium*, which is required for this phylum name (International Code of Nomenclature of Prokaryotes, ICNP rule 8), was already proposed by Lim *et al*. [27] and validated.

## Description of *Fidelibacter* gen. nov.

*Fidelibacter* (Fi.de.li.bac’ter. L. masc. adj. *fidelis*, faithful; N.L. masc. n. *bacter*, a rod; N.L. masc. n. *Fidelibacter*, a rod relying on other bacteria).

Obligately anaerobic, Gram-negative, non-motile, non-spore-forming, irregular rod cells. Rod cells change to a spherical shape in the absence of exogenous muropeptide. The major end products from yeast extract degradation are acetate, hydrogen and carbon dioxide. No anaerobic respiration with nitrate, nitrite, sulphate or Fe(III) is observed. The major cellular fatty acids are C16 : 0, C18 : 1 *trans*9, C18 : 0 and C17 : 0. Cell-wall murein contains alanine, glutamic acid and lysin. The DNA G+C content of the type species is 45.6 mol%. The type species is *F. multiformis*.

## Description of *F. multiformis* sp. nov.

*F. multiformis* (mul.ti.for’mis. L. masc. adj. *multiformis*, many-shaped, multiform).

Shows the following characteristics in addition to those given for the genus. Grows at 25–45 °C (optimally at 40 °C), at pH 6.8–8.5 (optimally at pH 7.8) and in the presence of 0.05–1.2 M NaCl (optimally in 0.3–0.4 M NaCl). Muropeptides and yeast extract are required for growth and serve as energy and carbon sources. d-Lactate is utilized. The following compounds do not support growth: pyruvate, glucose, fructose, galactose, mannose, ribose, xylose, arabinose, sucrose, lactose, starch, cellulose, mannitol, sorbitol, raffinose or cellobiose. In pure culture, the end products of muropeptides and yeast extract degradation are acetate, hydrogen and carbon dioxide. Peptone or casamino acids do not stimulate growth. Growth is enhanced in co-culture with a hydrogen-scavenging methanogen. Sensitive to chloramphenicol, vancomycin and rifampicin, but resistant to neomycin, kanamycin and ampicillin. Colonies are white, slightly irregular discs with a wavy margin on the deep agar slant. The type strain, IA91^T^ (=JCM 39387^T^=KCTC 25736^T^), was isolated from a slurry of sediments and formation water derived from a deep sedimentary, natural-gas-bearing saline aquifer in Japan. The GenBank/EMBL/DDBJ accession numbers for the 16S rRNA gene sequence and genome sequences of strain IA91^T^ are LC818858 and AP035449, respectively.

## Description of *Fidelibacteraceae* fam. nov.

*Fidelibacteraceae* (Fi.de.li.bac.te.ra.ce’ae. N.L. masc. n. *Fidelibacter*, type genus of the family; suff. -*aceae*, ending to denote a family; N.L. fem. pl. n. *Fidelibacteraceae,* the family of the genus *Fidelibacter*).

The description is the same as for the genus *Fidelibacter*. Type genus is *Fidelibacter*.

## Description of *Fidelibacterales* ord. nov.

*Fidelibacterales* (Fi.de.li.bac.te.ra’les. N.L. masc. n. *Fidelibacter*, type genus of the order; suff. -*ales*, ending to denote an order; N.L. fem. pl. n. *Fidelibacterales*, the order of the genus *Fidelibacter*).

The description is the same as for the genus *Fidelibacter*. Type genus is *Fidelibacter*.

## Description of *Fidelibacteria* classis nov.

*Fidelibacteria* (Fi.de.li.bac.te’ri.a. N.L. masc. n. *Fidelibacter*, type genus of the class; suff. -*ia*, ending to denote a class; N.L. neut. pl. n. *Fidelibacteria*, the class of the genus *Fidelibacter*).

The description is the same as for the genus *Fidelibacter*. Type genus is *Fidelibacter*.

## Description of *Fidelibacterota* Phyl. nov.

*Fidelibacterota* (Fi.de.li.bac.te.ro’ta. N.L. masc. n. *Fidelibacter*, type genus of the phylum; -*ota*, ending to denote a phylum; N.L. neut. pl. n. *Fidelibacterota*, the phylum of the genus *Fidelibacter*).

The phylum *Fidelibacterota* is defined based on phylogenetic and phylogenomic analyses of the sole isolated strain IA91^T^ and uncultured representatives from various environments. Type genus is *Fidelibacter*.

## supplementary material

10.1099/ijsem.0.006558 Uncited Supplementary Material 1.

10.1099/ijsem.0.006558 Fig. S4.

## Abbreviations

ANI: average nucleotide identity
GTDB: genome taxonomy database
MAG: metagenome-assembled genome
MG-A: Marine Group A
MurNAc: *N*-acetylmuramic acid
PG: peptidoglycan
