# Supplementary material for: Fidelibacter multiformis gen. nov., sp. nov., isolated from a deep subsurface aquifer and proposal of Fidelibacterota phyl. nov., formerly called Marine Group A, SAR406 or Candidatus Marinimicrobia
Source: Int J Syst Evol Microbiol. 2024 Oct 25;74(10):006558. doi: 10.1099/ijsem.0.006558 (PMC11652737; doi:10.1099/ijsem.0.006558)
Supplement: Uncited Supplementary Material 1. [file ijsem-74-06558-s001.pdf]

Supplementary material for

Fidelibacter multiformis gen. nov., sp. nov., isolated from a deep subsurface aquifer and proposal of *Fidelibacterota* phyl. nov., formerly called Marine Group A, SAR406 or *Ca*. Marinimicrobia

Taiki Katayama, Masaru K. Nobu, Yoichi Kamagata and Hideyuki Tamaki

Correspondence to: katayama.t@aist.go.jp

Supplementary Table S1. List of the conserved marker proteins used for phylogenetic analysis.

Accession number	Protein
PFAM_PF00380.20	ribosomal protein S9/S16
PFAM_PF00410.20	ribosomal protein S8
PFAM_PF00466.21	ribosomal protein L10
PFAM_PF01025.20	GrpE
PFAM_PF03726.15	Polyribonucleotide nucleotidyltransferase, RNA binding domain
TIGR_TIGR00006	16S rRNA (cytosine(1402)-N(4))-methyltransferase
TIGR_TIGR00019	peptide chain release factor 1
TIGR_TIGR00020	peptide chain release factor 2
TIGR_TIGR00029	ribosomal protein bS20
TIGR_TIGR00043	rRNA maturation RNase YbeY
TIGR_TIGR00059	ribosomal protein bL17
TIGR_TIGR00061	ribosomal protein bL21
TIGR_TIGR00082	ribosome-binding factor A
TIGR_TIGR00084	Holliday junction DNA helicase RuvA
TIGR_TIGR00086	SsrA-binding protein
TIGR_TIGR00088	tRNA (guanine(37)-N(1))-methyltransferase
TIGR_TIGR00090	ribosome silencing factor
TIGR_TIGR00092	GTP-binding protein YchF
TIGR_TIGR00095	16S rRNA (guanine(966)-N(2))-methyltransferase RsmD
TIGR_TIGR00115	trigger factor
TIGR_TIGR00116	translation elongation factor Ts
TIGR_TIGR00158	ribosomal protein bL9
TIGR_TIGR00166	ribosomal protein bS6
TIGR_TIGR00168	translation initiation factor IF-3
TIGR_TIGR00194	excinuclease ABC subunit C
TIGR_TIGR00250	putative transcription antitermination factor YqgF
TIGR_TIGR00344	alanine--tRNA ligase
TIGR_TIGR00362	chromosomal replication initiator protein DnaA
TIGR_TIGR00392	isoleucine--tRNA ligase
TIGR_TIGR00396	leucine--tRNA ligase
TIGR_TIGR00398	methionine--tRNA ligase
TIGR_TIGR00414	serine--tRNA ligase
TIGR_TIGR00420	tRNA (5-methylaminomethyl-2-thiouridylate)-methyltransferase
TIGR_TIGR00431	tRNA pseudouridine(55) synthase
TIGR_TIGR00435	cysteine--tRNA ligase
TIGR_TIGR00442	histidine--tRNA ligase
TIGR_TIGR00456	arginine--tRNA ligase
TIGR_TIGR00459	aspartate--tRNA ligase
TIGR_TIGR00460	methionyl-tRNA formyltransferase
TIGR_TIGR00468	phenylalanine--tRNA ligase, alpha subunit
TIGR_TIGR00472	phenylalanine--tRNA ligase, beta subunit
TIGR_TIGR00487	translation initiation factor IF-2
TIGR_TIGR00496	ribosome recycling factor
TIGR_TIGR00580	transcription-repair coupling factor
TIGR_TIGR00593	DNA polymerase I
TIGR_TIGR00615	recombination protein RecR
TIGR_TIGR00631	excinuclease ABC subunit B
TIGR_TIGR00634	DNA repair protein RecN
TIGR_TIGR00635	Holliday junction DNA helicase RuvB
TIGR_TIGR00643	ATP-dependent DNA helicase RecG
TIGR_TIGR00663	DNA polymerase III, beta subunit
TIGR_TIGR00717	ribosomal protein bS1
TIGR_TIGR00755	ribosomal RNA small subunit methyltransferase A
TIGR_TIGR00810	preprotein translocase, SecG subunit
TIGR_TIGR00922	transcription termination/antitermination factor NusG
TIGR_TIGR00959	signal recognition particle protein
TIGR_TIGR00963	preprotein translocase, SecA subunit
TIGR_TIGR00964	preprotein translocase, SecE subunit
TIGR_TIGR00967	preprotein translocase, SecY subunit
TIGR_TIGR01009	ribosomal protein uS3
TIGR_TIGR01011	ribosomal protein uS2
TIGR_TIGR01017	ribosomal protein uS4
TIGR_TIGR01021	ribosomal protein uS5
TIGR_TIGR01029	ribosomal protein uS7
TIGR_TIGR01032	ribosomal protein bL20
TIGR_TIGR01044	ribosomal protein uL22
TIGR_TIGR01059	DNA gyrase, B subunit
TIGR_TIGR01063	DNA gyrase, A subunit
TIGR_TIGR01066	ribosomal protein uL13
TIGR_TIGR01071	ribosomal protein uL15
TIGR_TIGR01079	ribosomal protein uL24
TIGR_TIGR01128	DNA polymerase III, delta subunit
TIGR_TIGR01164	ribosomal protein uL16
TIGR_TIGR01169	ribosomal protein uL1
TIGR_TIGR01171	ribosomal protein uL2
TIGR_TIGR01391	DNA primase
TIGR_TIGR01393	elongation factor 4
TIGR_TIGR01632	ribosomal protein uL11
TIGR_TIGR01951	transcription antitermination factor NusB
TIGR_TIGR01953	transcription termination factor NusA
TIGR_TIGR02012	protein RecA
TIGR_TIGR02013	DNA-directed RNA polymerase, beta subunit
TIGR_TIGR02027	DNA-directed RNA polymerase, alpha subunit
TIGR_TIGR02191	ribonuclease III
TIGR_TIGR02350	chaperone protein DnaK
TIGR_TIGR02386	DNA-directed RNA polymerase, beta' subunit
TIGR_TIGR02397	DNA polymerase III, subunit gamma and tau
TIGR_TIGR02432	tRNA(Ile)-lysidine synthetase
TIGR_TIGR03594	ribosome-associated GTPase EngA
TIGR_TIGR03625	50S ribosomal protein uL3
TIGR_TIGR03632	ribosomal protein uS11
TIGR_TIGR03654	ribosomal protein uL6
TIGR_TIGR03953	50S ribosomal protein uL4

Supplementary Table S2. List of the conserved marker proteins used for phylogenetic analysis.

Accession no.	Species	Phylum	ANI value (%)
GCA_001886815	*Caldithrix abyssi*	*Calditrichota*	68.2
GCA_024278715	*Caldithrix sp024278715*	*Calditrichota*	67.9
GCA_000020465	*Chlorobium limicola*	*Chlorobiota*	70.1
GCA_000020525	*Chloroherpeton thalassium*	*Chlorobiota*	69.1
GCA_000258405	*Ignavibacterium album*	*Ignavibacteriota*	68.0
GCA_000279145	*Melioribacter roseus*	*Ignavibacteriota*	68.9
GCA_000013045	*Salinibacter ruber*	*Bacteroidota*	69.4
GCA_000014145	*Cytophaga hutchinsonii*	*Bacteroidota*	69.2
GCA_000024005	*Chitinophaga pinensis*	*Bacteroidota*	69.5
GCA_000024525	*Spirosoma linguale*	*Bacteroidota*	68.9
GCA_000024845	*Rhodothermus marinus*	*Bacteroidota*	69.0
GCA_000025985	*Bacteroides fragilis*	*Bacteroidota*	69.9
GCA_000143765	*Sphingobacterium spiritivorum*	*Bacteroidota*	68.6
GCA_000169175	*Microscilla marina*	*Bacteroidota*	69.0
GCA_000183135	*Paludibacter propionicigenes*	*Bacteroidota*	68.8
GCA_000189415	*Weeksella virosa*	*Bacteroidota*	68.9
GCA_000205165	*Paraprevotella xylaniphila*	*Bacteroidota*	68.6
GCA_000212375	*Porphyromonas asaccharolytica*	*Bacteroidota*	69.7
GCA_000212735	*Haliscomenobacter hydrossis*	*Bacteroidota*	69.2
GCA_000222485	*Cyclobacterium marinum*	*Bacteroidota*	68.6
GCA_000233955	*Paraprevotella clara*	*Bacteroidota*	68.9
GCA_000259075	*Marinilabilia salmonicolor*	*Bacteroidota*	69.5
GCA_000375465	*Balneola vulgaris*	*Bacteroidota*	69.5
GCA_000380985	*Proteiniphilum acetatigenes*	*Bacteroidota*	70.0
GCA_000422585	*Hugenholtzia roseola*	*Bacteroidota*	66.8
GCA_000427365	*Rikenella microfusus*	*Bacteroidota*	69.5
GCA_000512915	*Barnesiella viscericola*	*Bacteroidota*	69.1
GCA_000621705	*Prolixibacter bellariivorans*	*Bacteroidota*	68.6
GCA_000709555	*Walczuchella monophlebidarum*	*Bacteroidota*	70.9
GCA_000757385	*Flavobacterium aquatile*	*Bacteroidota*	70.1
GCA_001006485	*Tannerella forsythia*	*Bacteroidota*	69.3
GCA_001192835	*Lentimicrobium saccharophilum*	*Bacteroidota*	68.9
GCA_001310955	*Marinifilum fragile*	*Bacteroidota*	69.2
GCA_001443605	*Salinivirga cyanobacteriivorans*	*Bacteroidota*	68.9
GCA_002201515	*Muribaculum intestinale*	*Bacteroidota*	68.4
GCA_002257665	*Rubricoccus marinus*	*Bacteroidota*	71.3
GCA_002369955	*Ichthyobacterium seriolicida*	*Bacteroidota*	74.3
GCA_003003005	*Mongoliibacter ruber*	*Bacteroidota*	68.1
GCA_003149185	*Sediminitomix flava*	*Bacteroidota*	69.0
GCA_003285105	*Roseithermus sacchariphilus*	*Bacteroidota*	68.4
GCA_003313335	*Pedobacter zeaxanthinifaciens*	*Bacteroidota*	71.7
GCA_003337435	*Schleiferia thermophila*	*Bacteroidota*	69.8
GCA_004340205	*Acetobacteroides hydrogenigenes*	*Bacteroidota*	68.5
GCA_007993035	*Vicingus serpentipes*	*Bacteroidota*	69.9
GCA_007995015	*Luteibaculum oceani*	*Bacteroidota*	70.7
GCA_008806325	*Salibacter halophilus*	*Bacteroidota*	70.1
GCA_010119975	*Rhodocytophaga rosea*	*Bacteroidota*	68.9
GCA_010686655	*Cryomorpha ignava*	*Bacteroidota*	69.6
GCA_011059145	*Halalkalibaculum roseum*	*Bacteroidota*	68.3
GCA_011682235	*Rhodocaloribacter litoris*	*Bacteroidota*	68.5
GCA_013106755	*Limnovirga soli*	*Bacteroidota*	68.6
GCA_014518315	*Taishania pollutisoli*	*Bacteroidota*	70.3
GCA_015476115	*Aegicerativicinus sediminis*	*Bacteroidota*	68.9
GCA_017921895	*Natronogracilivirga saccharolytica*	*Bacteroidota*	68.1
GCA_018711925	*Coprenecus pullicola*	*Bacteroidota*	68.3
GCA_018715595	*Caccoplasma merdavium*	*Bacteroidota*	67.8
GCA_019114015	*Onthomorpha intestinigallinarum*	*Bacteroidota*	67.1
GCA_019130065	*Pinibacter aurantiacus*	*Bacteroidota*	68.8
GCA_020149915	*Sulfidibacterium hydrothermale*	*Bacteroidota*	68.8
GCA_030296935	*Coprobacter fastidiosus*	*Bacteroidota*	69.4
GCA_030503435	*Rhodocytophaga aerolata*	*Bacteroidota*	68.4
GCA_900070205	*Kryptonium mobile*	*Bacteroidota*	68.0
GCA_900096565	*Williamwhitmania taraxaci*	*Bacteroidota*	67.6
GCA_900104655	*Rikenella massiliensis*	*Bacteroidota*	69.8
GCA_900113045	*Thermoflexibacter ruber*	*Bacteroidota*	69.3
GCA_900114265	*Flaviramulus basaltis*	*Bacteroidota*	69.4
GCA_900176135	*Hymenobacter roseosalivarius*	*Bacteroidota*	68.1
GCA_907165195	*Parvicella tangerina*	*Bacteroidota*	71.6
GCA_000024665	*Fibrobacter succinogenes*	*Fibrobacterota*	69.5
GCA_000146505	*Fibrobacter succinogenes*	*Fibrobacterota*	69.5
GCA_000474745	*Chitinivibrio alkaliphilus*	*Fibrobacterota*	70.6
GCA_001045525	*Chitinispirillum alkaliphilum*	*Fibrobacterota*	68.8
GCA_001462245	*Fibromonas termitidis*	*Fibrobacterota*	66.4
GCA_002797675	*Hallerella succinigenes*	*Fibrobacterota*	69.2
GCA_003148885	*Hallerella porci*	*Fibrobacterota*	70.2
GCA_900167415	*Hallerella intestinalis*	*Fibrobacterota*	70.2

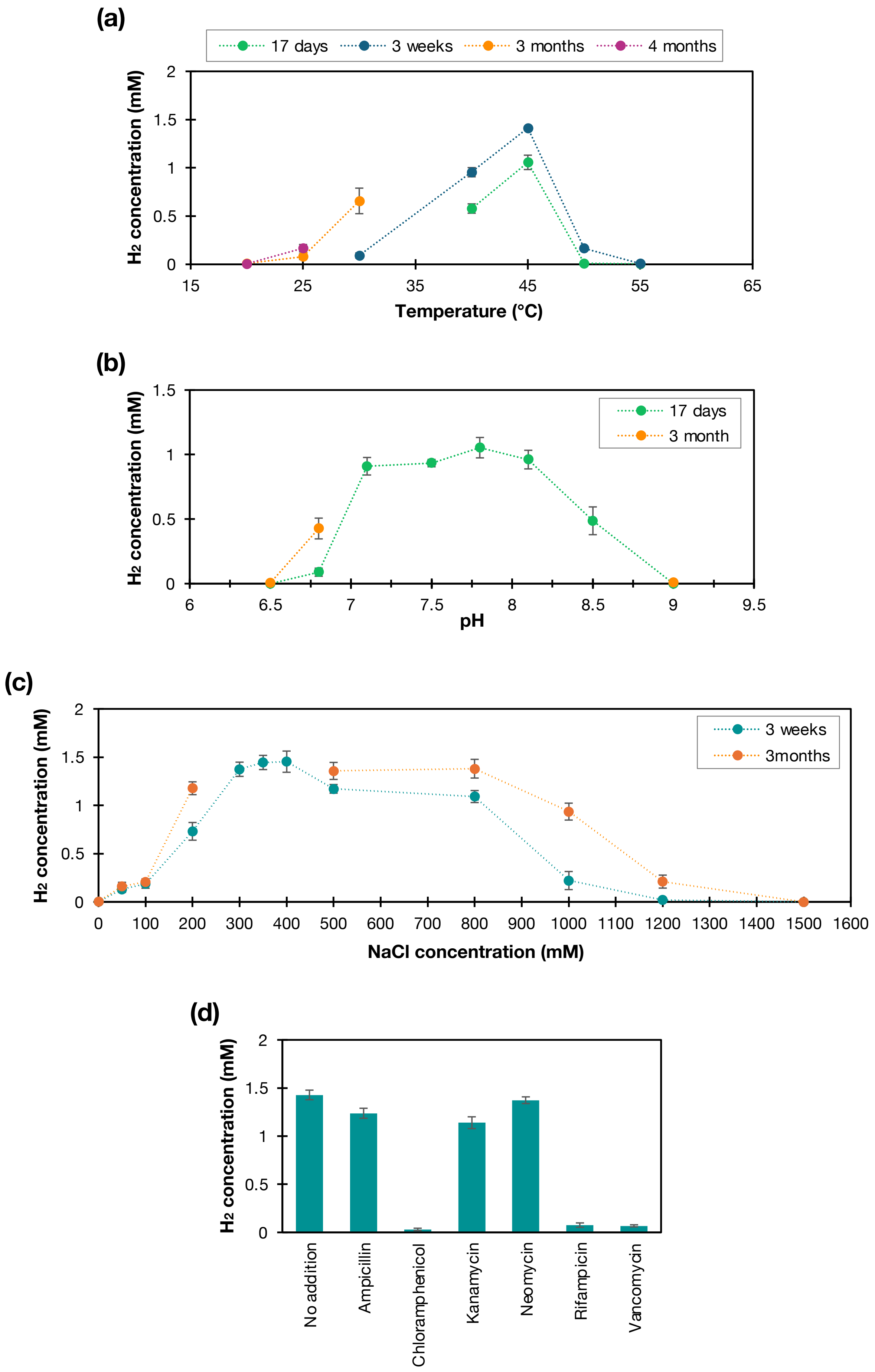

Supplementary Fig. S1. Effects of temperature (**a**), pH (**b**), salinity (**c**) and antibiotics (**d**) on IA91^T growth. H_2 production in anoxic culture of IA91^T was measured after up to 4 months (**a-c**) or 3 weeks (**d**) of incubation. Means and standard deviation (error bars) of triplicate cultures are shown.

(a)

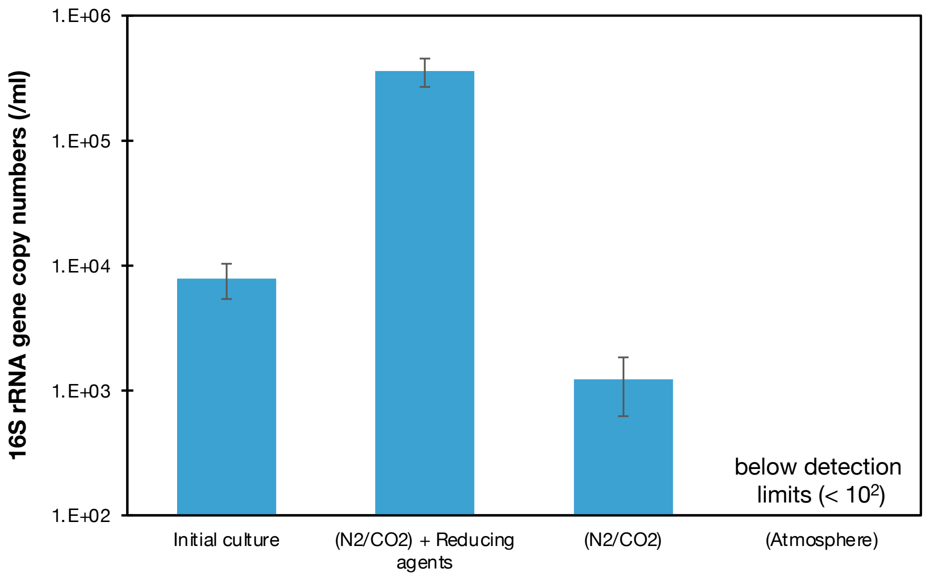

(b)

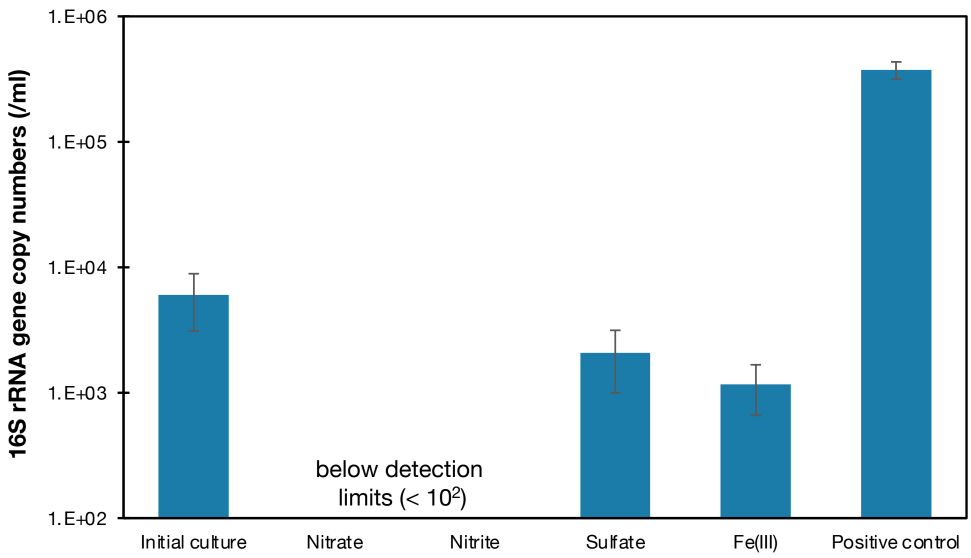

Supplementary Fig. S2. Oxygen tolerance (**a**) and anaerobic respiration (**b**) of IA91^T. The 16S rRNA gene copies numbers were measured before and after (1 month) incubation. Means and standard deviation (error bars) of triplicate cultures are shown. The culture supplemented with 0.5% yeast extract, autoclaved Acc8 culture supernatant and 8 mM D-lactate was used as a positive control (**b**).

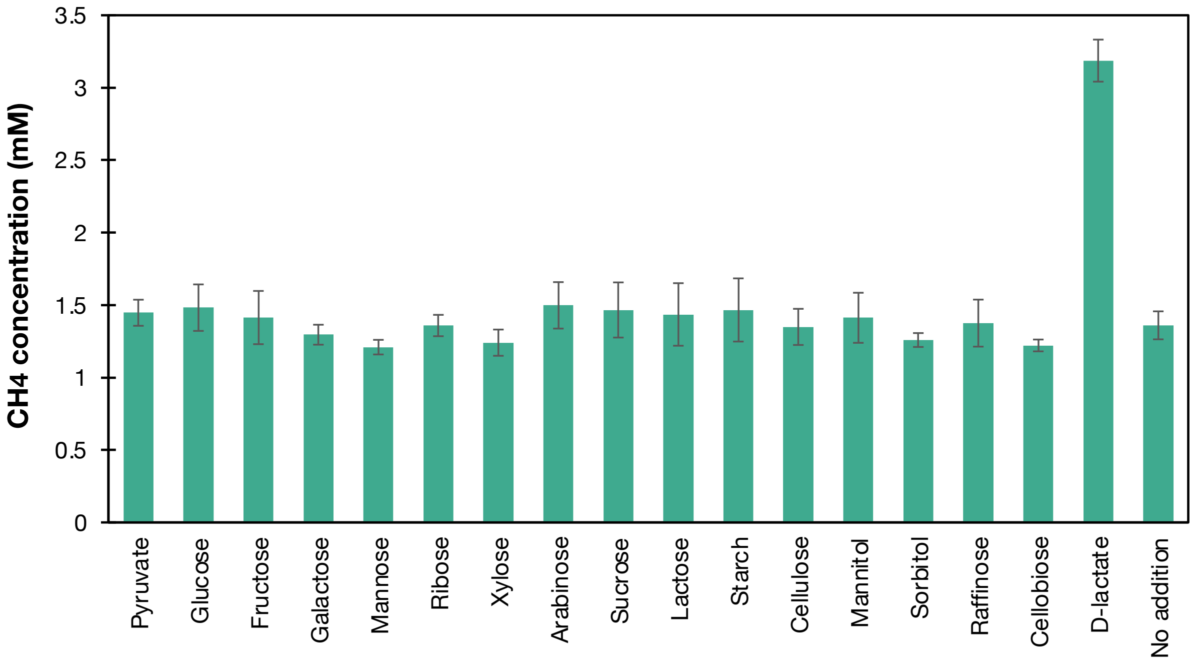

Supplementary Fig. S3. Substrate utilization of IA91^{T}. Means and standard deviation (error bars) of triplicate cultures are shown.

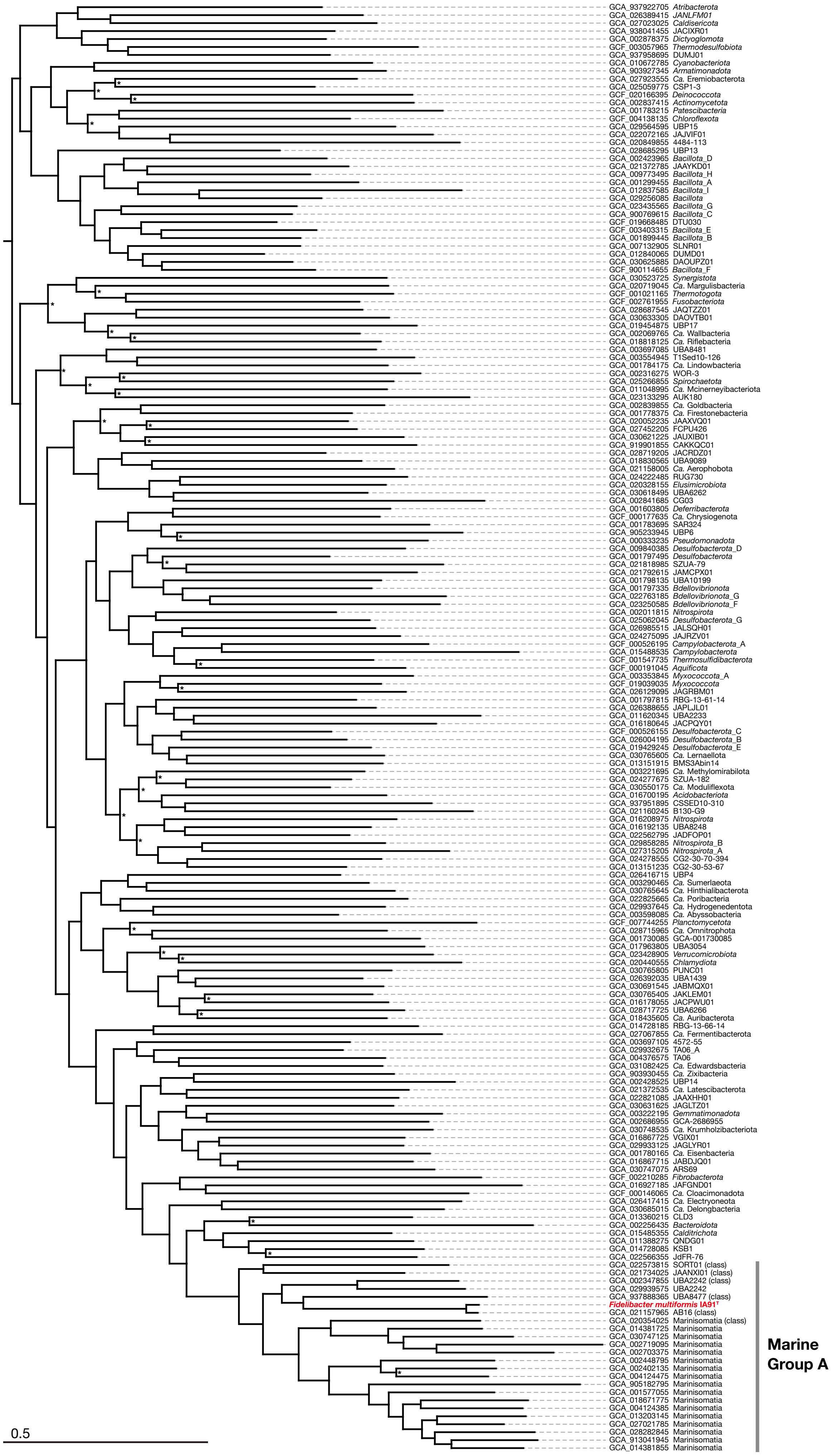
GCA_937922705 Atribacterota
GCA_026389415 JANLFM01
GCA_027023025 Caldisericota
GCA_938041455 JACIXR01
GCA_002878375 Dictyoglomota
GCF_003057965 Thermodesulfobiota
GCA_937958695 DUMJ01
GCA_010672785 Cyanobacteriota
GCA_903927345 Armatimonadota
GCA_027923555 Ca. Eremiobacterota
GCA_025059775 CSP1-3
GCF_020166395 Deinococcota
GCA_002837415 Actinomycetota
GCA_001783215 Patescibacteria
GCF_004138135 Chloroflexota
GCA_029564595 UBP15
GCA_022072165 JAJVIF01
GCA_020849855 4484-113
GCA_028685295 UBP13
GCA_002423965 Bacillota_D
GCA_021372785 JAAYKD01
GCA_009773495 Bacillota_H
GCA_001299455 Bacillota_A
GCA_012837585 Bacillota_I
GCA_029256085 Bacillota
GCA_023435565 Bacillota_G
GCA_900769615 Bacillota_C
GCF_019668485 DTU030
GCF_003403315 Bacillota_E
GCA_001899445 Bacillota_B
GCA_007132905 SLNR01
GCA_012840065 DUMD01
GCA_030625885 DAOUPZ01
GCF_900114655 Bacillota_F
GCA_030523725 Synergistota
GCA_020719045 Ca. Margulisbacteria
GCF_001021165 Thermotogota
GCF_002761955 Fusobacteriota
GCA_028687545 JAQTZZ01
GCA_030633305 DAOVTB01
GCA_019454875 UBP17
GCA_002069765 Ca. Wallbacteria
GCA_018818125 Ca. Riflebacteria
GCA_003697085 UBA8481
GCA_003554945 T1Sed10-126
GCA_001784175 Ca. Lindowbacteria
GCA_002316275 WOR-3
GCA_025266855 Spirochaetota
GCA_011048995 Ca. Mcinerneyibacteriota
GCA_023133295 AUK180
GCA_002839855 Ca. Goldbacteria
GCA_001778375 Ca. Firestonebacteria
GCA_020052235 JAAXVQ01
GCA_027452205 FCPU426
GCA_030621225 JAUXIB01
GCA_919901855 CAKKQC01
GCA_028719205 JACRDZ01
GCA_018830565 UBA9089
GCA_021158005 Ca. Aerophobota
GCA_024222485 RUG730
GCA_020328155 Elusimicrobiota
GCA_030618495 UBA6262
GCA_002841685 CG03
GCA_001603805 Deferribacterota
GCF_000177635 Ca. Chrysiogenota
GCA_001783695 SAR324
GCA_905233945 UBP6
GCA_000333235 Pseudomonadota
GCA_009840385 Desulfobacterota_D
GCA_001797495 Desulfobacterota
GCA_021818985 SZUA-79
GCA_021792615 JAMCPX01
GCA_001798135 UBA10199
GCA_001797335 Bdellovibrionota
GCA_022763185 Bdellovibrionota_G
GCA_023250585 Bdellovibrionota_F
GCA_002011815 Nitrospirota
GCA_025062045 Desulfobacterota_G
GCA_026985515 JALSQH01
GCA_024275095 JAJRZV01
GCF_000526195 Campylobacterota_A
GCA_015488535 Campylobacterota
GCF_001547735 Thermosulfidibacterota
GCF_000191045 Aquificota
GCA_003353845 Myxococcota_A
GCF_019039035 Myxococcota
GCA_026129095 JAGRBM01
GCA_001797815 RBG-13-61-14
GCA_026388655 JAPLJL01
GCA_011620345 UBA2233
GCA_016180645 JACPQY01
GCF_000526155 Desulfobacterota_C
GCA_026004195 Desulfobacterota_B
GCA_019429245 Desulfobacterota_E
GCA_030765605 Ca. Lernaellota
GCA_013151915 BMS3Abin14
GCA_003221695 Ca. Methylomirabilota
GCA_024277675 SZUA-182
GCA_030550175 Ca. Moduliflexota
GCA_016700195 Acidobacteriota
GCA_937951895 CSSED10-310
GCA_021160245 B130-G9
GCA_016208975 Nitrospirota
GCA_016192135 UBA8248
GCA_022562795 JADFOP01
GCA_029858285 Nitrospirota_B
GCA_027315205 Nitrospirota_A
GCA_024278555 CG2-30-70-394
GCA_013151235 CG2-30-53-67
GCA_026416715 UBP4
GCA_003290465 Ca. Sumerlaeota
GCA_030765645 Ca. Hinthialibacterota
GCA_022825665 Ca. Poribacteria
GCA_029937645 Ca. Hydrogenedentota
GCA_003598085 Ca. Abyssobacteria
GCF_007744255 Planctomycetota
GCA_028715965 Ca. Omnitrophota
GCA_001730085 GCA-001730085
GCA_017963805 UBA3054
GCA_023428905 Verrucomicrobiota
GCA_020440555 Chlamydiota
GCA_030765805 PUNC01
GCA_026392035 UBA1439
GCA_030691545 JABMQX01
GCA_030765405 JAKLEM01
GCA_016178055 JACPWU01
GCA_028717725 UBA6266
GCA_018435605 Ca. Auribacterota
GCA_014728185 RBG-13-66-14
GCA_027067855 Ca. Fermentibacterota
GCA_003697105 4572-55
GCA_029932675 TA06_A
GCA_004376575 TA06
GCA_031082425 Ca. Edwardsbacteria
GCA_903930455 Ca. Zixibacteria
GCA_002428525 UBP14
GCA_021372535 Ca. Latescibacterota
GCA_022821085 JAAXHH01
GCA_030631625 JAGLTZ01
GCA_003222195 Gemmatimonadota
GCA_002686955 GCA-2686955
GCA_030748535 Ca. Krumholzibacteriota
GCA_016867725 VGIX01
GCA_029933125 JAGLYR01
GCA_001780165 Ca. Eisenbacteria
GCA_016867715 JABDJQ01
GCA_030747075 ARS69
GCF_002210285 Fibrobacterota
GCA_016927185 JAFGND01
GCF_000146065 Ca. Cloacimonadota
GCA_026417415 Ca. Electryoneota
GCA_030685015 Ca. Delongbacteria
GCA_013360215 CLD3
GCA_002256435 Bacteroidota
GCA_015485355 Calditrichota
GCA_011388275 QNDG01
GCA_014728085 KSB1
GCA_022566355 JdFR-76
GCA_022573815 SORT01 (class)
GCA_021734025 JAANXI01 (class)
GCA_002347855 UBA2242 (class)
GCA_029939575 UBA2242
GCA_937888365 UBA8477 (class)
Fidelibacter multiformis IA91ᵀ
GCA_021157965 AB16 (class)
GCA_020354025 Marinisomatia (class)
GCA_014381725 Marinisomatia
GCA_030747125 Marinisomatia
GCA_002719095 Marinisomatia
GCA_002703375 Marinisomatia
GCA_002448795 Marinisomatia
GCA_002402135 Marinisomatia
GCA_004124475 Marinisomatia
GCA_905182795 Marinisomatia
GCA_001577055 Marinisomatia
GCA_018671775 Marinisomatia
GCA_004124385 Marinisomatia
GCA_013203145 Marinisomatia
GCA_027021785 Marinisomatia
GCA_028282845 Marinisomatia
GCA_913041945 Marinisomatia
GCA_014381855 Marinisomatia
Marine
Group A
0.5

Supplementary Fig. S4. A midpoint-rooted maximum-likelihood tree showing the relationship of strain IA91^T (bold red), MG-A members, and other cultured and uncultured bacterial phyla recognized in GTDB based on conserved marker protein sequences involved in replication, transcription and translation. Branches with lower bootstrap values (<95%) are indicated by asterisk.
